# Fatness mediates the influence of muscular fitness on metabolic syndrome in Colombian collegiate students

**DOI:** 10.1371/journal.pone.0173932

**Published:** 2017-03-15

**Authors:** Antonio García-Hermoso, Hugo Alejandro Carrillo, Katherine González-Ruíz, Andrés Vivas, Héctor Reynaldo Triana-Reina, Javier Martínez-Torres, Daniel Humberto Prieto-Benavidez, Jorge Enrique Correa-Bautista, Jeison Alexander Ramos-Sepúlveda, Emilio Villa-González, Mark D. Peterson, Robinson Ramírez-Vélez

**Affiliations:** 1 Laboratorio de Ciencias de la Actividad Física, el Deporte y la Salud, Facultad de Ciencias Médicas, Universidad de Santiago de Chile, USACH, Santiago, Chile; 2 Grupo GRINDER, programa de Educación Física y Deportes, Universidad del Valle, Santiago de Cali, Colombia; 3 Grupo de Ejercicio Físico y Deportes, Vicerrectoría de Investigaciones, Universidad Manuela Beltrán, Bogotá DC, Colombia; 4 Grupo GICAEDS, Facultad de Cultura Física, Deporte y Recreación, Universidad Santo Tomás, Bogotá DC, Colombia; 5 Centro de Estudios para la Medición de la Actividad Física «CEMA», Escuela de Medicina y Ciencias de la Salud, Universidad del Rosario, Bogotá DC, Colombia; 6 Facultad de Educación a Distancia y Virtual. Institución Universitaria Antonio José Camacho, Santiago de Cali, Colombia; 7 PROFITH ‘‘PROmoting FITness and Health through physical activity” research group, Department of Physical Education and Sport, School of Sport Sciences, University of Granada, Granada, Spain; 8 Department of Physical Medicine and Rehabilitation, University of Michigan, Ann Arbor, Michigan, United States of America; 9 Global REACH, University of Michigan Medical School, University of Michigan, Ann Arbor, Michigan, United States of America; John Hopkins University School of Medicine, UNITED STATES

## Abstract

The purpose of this study was two-fold: to analyze the association between muscular fitness (MF) and clustering of metabolic syndrome (MetS) components, and to determine if fatness parameters mediate the association between MF and MetS clustering in Colombian collegiate students. This cross-sectional study included a total of 886 (51.9% women) healthy collegiate students (21.4 ± 3.3 years old). Standing broad jump and isometric handgrip dynamometry were used as indicators of lower and upper body MF, respectively. Also, a MF score was computed by summing the standardized values of both tests, and used to classify adults as fit or unfit. We also assessed fat mass, body mass index, waist-to-height ratio, and abdominal visceral fat, and categorized individuals as low and high fat using international cut-offs. A MetS cluster score was derived by calculating the sum of the sample-specific z-scores from the triglycerides, HDL cholesterol, fasting glucose, waist circumference, and arterial blood pressure. Linear regression models were used to examine whether the association between MF and MetS cluster was mediated by the fatness parameters. Data were collected from 2013 to 2016 and the analysis was done in 2016. Findings revealed that the best profiles (fit + low fat) were associated with lower levels of the MetS clustering (p <0.001 in the four fatness parameters), compared with unfit and fat (unfit + high fat) counterparts. Linear regression models indicated a partial mediating effect for fatness parameters in the association of MF with MetS clustering. Our findings indicate that efforts to improve MF in young adults may decrease MetS risk partially through an indirect effect on improvements to adiposity levels. Thus, weight reduction should be taken into account as a complementary goal to improvements in MF within exercise programs.

## Introduction

International organizations such as the American College for Sports Medicine and American Heart Association encourage regular bone-strengthening and muscle-strengthening activities on at least 3 days per week [1]-a recommendation that is vital in the adult population given the inverse relation between muscular fitness (MF) and mortality [2, 3]. Studies with predominantly middle-aged and elderly individuals show that low hand-grip strength is associated with cardiovascular disease (CVD) [4]. Low levels of MF is also associated with age-related weight gains, hypertension, and prevalence of the metabolic syndrome (MetS) [5].

Assessments for MF, such as the standing broad jump or hand-grip strength, tend to be associated with lower fatness and better cardiovascular health in adult [6] and adolescent populations [7]. The association of fatness with lower MF is generally strongest when adiposity is measured through imaging techniques, slightly weaker when assessed using waist circumference (WC), body mass index (BMI) or other anthropometric estimates of body fat mass [8]. In contrast, high MF levels have been shown to reduce disease-related metabolic risk, such as oxidized-LDL and C-reactive protein levels in adults with excess adiposity [9].

Given that MF is inversely associated with fatness parameters [10] and that these predict several individual risk factors of the MetS [11], it is possible that the association between MF and MetS clustering may be mediated by fatness parameters. Scientific evidence indicates an independent association between MF and cardiovascular health in adults independently of fatness [12]. We have recently [6] demonstrated the importance of normalizing of MF to body mass for predicting cardiometabolic risk in a large sample of university students. However, few studies have investigated the independent contribution of MF and fatness parameters to individual and clustered MetS components. These studies show that fatness parameters could mediate the association between muscular fitness and MetS [13].

Studies examining the association between MF and MetS often incorporate multiple linear regression, logistic regression, or analysis of the covariance to adjust for confounding or mediator variables; however, these multivariate methods do not account for the percentage of the total effect explained by the potential mediators. Mediation analysis is a statistical method that can be used to elucidate the processes underlying an association between two variables and to understand the extent to which the association can be modified, mediated, or confounded by a third variable [14].

Obesity and physical inactivity are primary, interdependent CVD risk factors among Hispanic/Latino adults, raising concerns about whether an increased risk of these conditions also is manifested at younger ages (1–4). Previous research has demonstrated an independent association between muscle weakness and increased cardiometabolic risk factors (4). Therefore, describing the magnitude of these risk factors in young adults is important for prioritizing prevention and public health efforts. Identifying Latin American collegiate students who have high levels of fatness, but are fit, or conversely who have healthy body composition but are unfit, is of high public health importance. Nevertheless, there have been no studies to date to determine the association between MF and MetS cluster in Latin American collegiate students.

Therefore, the purpose of this study was two-fold: to analyze the association between MF and clustering of MetS components, and to determine the mediating influence of adiposity parameters on the association between MF and MetS clustering in collegiate students from Colombia.

## Materials and methods

### Study sample

We conducted the cross-sectional component of the FUPRECOL study (Association between Muscular Strength and Metabolic Risk Factors in Colombia) in Bogota, Colombia during the 2013-2016-college year. The analysis was done in 2016. A convenience sample comprised 886 volunteers (51.9% female, mean age = 21.4 years (3.3) years old) between the ages of 18 and 35 years of low to middle socioeconomic status (SES: 1–4 on a scale of 1–6 defined by the Colombian government), and enrolled in public or private university in the capital district of Bogota and Santiago de Cali, Colombia. Students were informed that their participation was voluntary with no penalty for not participating. Inclusion criteria were: no self-reported history of inflammatory joint disease or neurological disorders; and not an athlete participating at an elite level of sport. Volunteers were not compensated for their participation. Subjects with a medical or clinical diagnosis of a major systemic disease (including malignant conditions such as cancer), type 1 or 2 diabetes, high blood pressure, hypothyroidism/hyperthyroidism, a history of drug, regular use of multivitamins or inflammatory (trauma, contusions), infectious conditions, or ≥ 35 kg/m^2^ BMI were also excluded from the study. The University of Manuel Beltran (Colombia) Institutional Review Board in accordance with the latest version of the Declaration of Helsinki approved the study (UMB No 01-1802-2013). After reading and signing an informed consent to participate in the study, volunteers were given an appointment for a testing session at the University laboratories. The students who agreed to participate and who had signed the informed consent form were given appointments for the following procedures.

### Measures

#### Physical exam and fatness parameters

Each person was asked to answer questions related to a health survey, and their answers regarding sociodemographic data and personal and family medical history were recorded. After completing a questionnaire of general information, participants were instructed to wear shorts, a t-shirt, and remove any metal and jewelry. The body weight of the subjects was measured when the subjects were in their underwear and barefoot, on electronic scales (Model Tanita^®^ BC 420MA Tokyo, Japan). The height of the subjects was measured using a mechanical stadiometer platform (Seca^®^ 274, Hamburg, Germany). We calculated BMI (weight/height^2^) from the height (kg) and weight (m) measurements. Weight status was determined according to WHO criteria for overweight (BMI ≥25 kg/m^2^) and obesity (BMI ≥30 kg/m^2^) and [15]. The WC (cm) was measured as the narrowest point between the lower costal border and the iliac crest; in the cases where this was not evident, it was measured at the midpoint between the last rib and the iliac crest, using a tape measure (Ohaus^®^ 8004-MA, New Jersey, USA).

Lean mass (kg), body fat percentage (%) and visceral adipose tissue (VAT) (mm) were determined for bioelectrical impedance analysis (BIA) by a tetrapolar whole body impedance (Model Seca^®^ mBCA 514 Medical Body Composition Analyze, Hamburg, Germany). The VAT area was estimated, using a multiple regression equation including age, sex, anthropometric data and body composition, as previously described [16]. Testing was scheduled to allow for a 2-hour fasting window and participants were asked to void their bladder before testing to optimize muscle mass assessment accuracy. Subjects’ feet were guided onto the BIA foot sensors by the raters to ensure optimal contact and centralized heel placement. All BIA measurements were completed by a trained investigator according to the device manufacturers’ instructions. For the calculation of intra-inter observer TEM, at least 50 subjects needed to be measured (30 men, 20 women, aged 22.3 ± 2.1 years). The corresponding intra-observer technical error (% reliability) of the measurements was 95%. Macias et al. [16] proposed a surrogate cut-off to body fatness unhealthy (30% in men and 44% in women) and cut-off to VAT unhealthy (>10 mm in both sexes) to identify individuals at risk of excess adiposity, respectively [17].

#### Musculoskeletal fitness

The MF protocols used were appropriate for use in this age group and have acceptable levels of validity and reliability for the handgrip strength [18] and standing broad jump [19]. We used the standing broad jump and isometric handgrip dynamometry as indicators of lower and upper body MF, respectively. For the standing broad jump, subjects were instructed to jump as far as possible using a two footed take-off and landing technique. They were encouraged to use a countermovement to flex and then extend their knees, ankles, and hips and to swing their arms to maximise performance. Standing broad jump performance was calculated as the distance between the toes at take-off to the heels at the landing point. The best score from two correctly performed jumps was used for the analysis [18]. Handgrip strength was assessed as an indicator of upper-body MF using an adjustable analogue handgrip dynamometer T-18 TKK SMEDLY III^®^ (Takei Scientific Instruments Co., Ltd, Niigata, Japan). Participants watched a brief demonstration of technique and were given verbal instructions on how to perform the test. The dynamometer was adjusted according to the subjects’ hand size according to a predetermined protocol [18]. The best score for each hand was recorded in kilograms. The handgrip strength (kg) was calculated as the average of the left and right arm. A MF score was computed by summing up the standardized values of standing broad jump and hand-grip strength. Several studies reveal that the least fit quartile showed the strongest association with a poor cardiometabolic risk profile and mortality [6, 19, 20], suggesting this could be considered the unfit group. Based on the mentioned approach, we conservatively considered the unfit group to be the age-and-sex-specific quartile 1 from our sample.

#### Clustering of Metabolic Syndrome (MetS) risk

After fasting for 12 hours, blood samples were obtained from a capillary sample at 6:30AM–7: 00AM, and prior to the fitness tests. Participants were asked to not engage in any prolonged exercise for the 24 hours prior to testing. The biochemical profile included: 1) the plasma lipid triglycerides (TG), total cholesterol, high-density lipoprotein cholesterol (HDL-c), fasting glucose, and low-density lipoprotein cholesterol (LDL-c) (by enzymatic colorimetric methods). Inter-assay reproducibility (coefficient of variation) was determined from 80 replicate analyses of 8 plasma pools over 15 days, and shown to be 2.6%, 2.0%, 3.2%, 3.6% for TG, total cholesterol, HDL-c and LDL-c, respectively and 1.5% for fasting glucose. Blood pressure was measured twice from the left arm via an Omron M6 Comfort (Omron^®^ Healthcare Europe B.V., Hoofddorp, the Netherlands) while the participants were sitting still. The blood pressure monitor cuff was placed two to three finger widths above the bend of the arm and a two-minute pause was allowed between the first and the second measurements. The mean arterial pressure (MAP) was calculated using the following formula: MAP = (systolic blood pressure + (2 x diastolic blood pressure)) / 3.

We calculated a MetS cluster that reflects a continuous score of the five MetS risk factors. The MetS cluster was calculated from the individual subject’s data, based on the International Diabetes Federation (IDF) [21], and standard deviations using data from the entire subject cohort at baseline. The equation used was: MetS cluster = ([HDL-C: 40 *or* 50 mg/dL]/ SD) + ([TG: 150 mg/dL]/SD)+([fasting glucose: 100 mg/dL]/SD) + ([WC: 94 *or* 80 cm]/SD) + ([MAP: 100]/SD). The mean of this continuously distributed metabolic composite index was therefore zero by definition. The Institutional Ethics Committee in accordance with the latest version of the Declaration of Helsinki approved the study (UMB N° 01-1802-2013). After reading and signing an informed consent to participate in the study, volunteers were given an appointment for a testing session at the University laboratories. The students who agreed to participate and who had signed the informed consent form were given appointments for the procedures.

### Covariates

A standardized questionnaire was used to collect comprehensive information of substance use via a personal interview with participants [22]. Alcohol drinkers and tobacco smokers were defined, respectively, as subjects who had consumed any alcoholic beverage ≥1 times per week, and those who had smoked ≥10 cigarettes per week, at least 6 months. The accuracy of information on substance use obtained from questionnaires has been validated by different experiments and described in detail in our previous work [23].

### Statistical analyses

Statistical analyses were performed using Statistical Package for the Social Sciences software for Windows version 21.0 (IBM Corporation, New York). Distributions were determined by the Kolmogorov–Smirnov test. *P* values < 0.05 were considered significant. Independent *t-*tests *or* chi squared were applied to compare unadjusted means or frequencies by sex, respectively. To study the combined effects of MF and fatness parameters with the MetS cluster, we created diverse groups. MF was categorized as “fit” (2^nd^– 4^th^ quartile) and “unfit” (1^st^ quartile). Adiposity parameters were categorized as “high fat” and “low fat” using specific cut-offs for each parameter as previously described. Four groups were used: (i) unfit and high fat; (ii) unfit and low fat; (iii) fit and high fat; and (iv) fit and low fat. To examine the differences between groups, we performed ANCOVA with age, gender, lean mass, alcohol and tobacco intake, and geographical area included as covariates. *Post-hoc* analyses with the Bonferroni’s correction were used to check the differences across groups.

The test of multicollinearity was done before analyzing mediation models. The tolerance value and variance inflation factors value appeared normal with values ranging between 0.273 and 0.567 for collinearity tolerance [24] and 1.763 and 3.689 for collinearity [25]. To examine whether the association between MF and MetS cluster was mediated by fatness parameters, linear regression models were fitted using bootstrapped mediation procedures included in the PROCESS SPSS script [26], adjusted for age, gender, lean mass, alcohol and tobacco intake, and geographical area. This script used bootstrapping methods recommended by Preacher and Hayes [27] for testing mediation hypotheses, using a resampling procedure of 10,000 bootstrap samples.

The first equation regressed the mediator (FM, BMI, or VAT) on the independent variable (MF). The second equation regressed the dependent variable (MetS cluster) on the independent variable (MF). The third equation regressed the dependent variable (MetS cluster) on both the independent (MF) and the mediator variable (FM, BMI, or VAT). The following criteria were used to establish if there is a significant indirect effect (“mediation”): (1) the independent variable (MF) is significantly related to the mediator (FM, BMI, or VAT); (2) the independent variable (MF) is significantly related to the dependent variable (MetS cluster); (3) the mediator (FM, BMI, or VAT) is significantly related to the dependent variable (MetS cluster); and (4) the association between the independent and dependent variable (i.e., the “direct effect”) is attenuated when the mediator is included in the regression model. The Sobel test is conducted by comparing the strength of the indirect effect of X (independent variable) on Y (dependent variable) to the point null hypothesis that it equals zero [28]. This analysis was adjusted by age, gender and lean mass. The level of statistical significance was established as p<0.05.

## Results

Table 1 shows the characteristics of the sample. The final sample had a mean age (standard deviation [SD]; [range]) of 21.4 years (3.3; [19–23]) and contained slightly more women (51.9%), compared to men (48.1%). Women had lower levels of body mass, height, WC, lean mass, blood pressure, hand-grip, standing broad jump, and TG than men (p< 0.05).

**Table 1:** Characteristics of the participants, by sex.

Characteristics	All participants	Men	Women	P value
	*n* = 426	*n* = 426	*n* = 460	
Age (years)	21.4 (3.3)	21.3 (3.4)	21.5 (3.2)	0.478
Body mass (kg)	64.2 (12.5)	69.9 (12.5)	58.9 (10.0)	<0.001
Height (m)	1.66 (0.11)	1.72 (0.07)	1.60 (0.10)	<0.001
BMI (kg/m^2^)	23.2 (3.7)	23.5 (3.7)	23.0 (3.7)	0.097
WC (cm)	75.4 (9.6)	79.1 (9.8)	72.0 (8.1)	<0.001
VAT (mm)	2.5 (2.4)	3.0 (2.9)	2.0 (1.6)	<0.001
Fat mass (%)	21.6 (8.8)	16.0 (6.7)	26.8 (7.2)	<0.001
Lean mass (kg)	49.7 (9.8)	58.5 (7.3)	42.6 (4.2)	<0.001
SBP (mmHg)	117.9 (12.7)	123.7 (11.7)	112.6 (11.1)	<0.001
DBP (mmHg)	74.3 (10.4)	76.8 (10.8)	72.1 (9.5)	<0.001
MBP (mmHg)	88.8 (9.9)	92.4 (9.7)	85.5 (8.9)	<0.001
Total cholesterol (mg/dL)	142.4 (33.6)	135.5 (31.3)	148.7 (34.5)	<0.001
Triglycerides (mg/dL)	95.5 (49.2)	99.0 (51.0)	92.2 (47.2)	0.040
LDL-c (mg/dL)	84.1 (27.3)	81.7 (26.5)	86.1 (27.8)	<0.001
HDL-c (mg/dL)	44.0 (12.8)	39.8 (10.7)	47.8 (13.4)	<0.001
Glucose (mg/dL)	83.3 (13.7)	82.7 (13.0)	83.9 (14.2)	0.179
MetS cluster	0.19 (2.82)	0.24 (3.01)	0.14 (2.63)	0.619
Hand-grip (kg)	32.0 (9.9)	40.0 (7.1)	24.5 (5.1)	<0.001
Hand-grip (z-score)	-0.019 (0.979)	-0.031 (0.972)	-0.008 (0.987)	0.726
Standing broad jump (cm)	150.6 (40.6)	182.6 (33.5)	124.3 (23.5)	<0.001
Standing broad jump (z-score)	-0.005 (0.981)	-0.012 (0.978)	0.001 (0.984)	0.872
Muscular fitness	-0.0233 (1.430)	-0.0390 (1.446)	-0.0089 (1.417)	0.754
Tobacco (≥10 cigarettes per week), n [%]*	60 [6.7]	30 [8.9]	22 [4.7]	0.349
Alcohol (≥1 times per week), %, n [%]*	92 [10.3]	50 [11.7]	42 [9.1]	0.041

Values are mean (SD) *or* [frequencies]

Note: Boldface indicates statistical significance. BMI: Body Mass Index; WC: Waist circumference; VAT: visceral fat; SBP: Systolic blood pressure; DBP: Diastolic blood pressure; MBP: Mean blood pressure; Muscular fitness, which is an average z-score computed from the hand-grip z-score and the SLJ z-score; MetS cluster was calculated for continuous z-score of the five metabolic syndrome factors. *t-*test or chi squared* was used to examine any significant difference by sex group.

**Fig 1:**
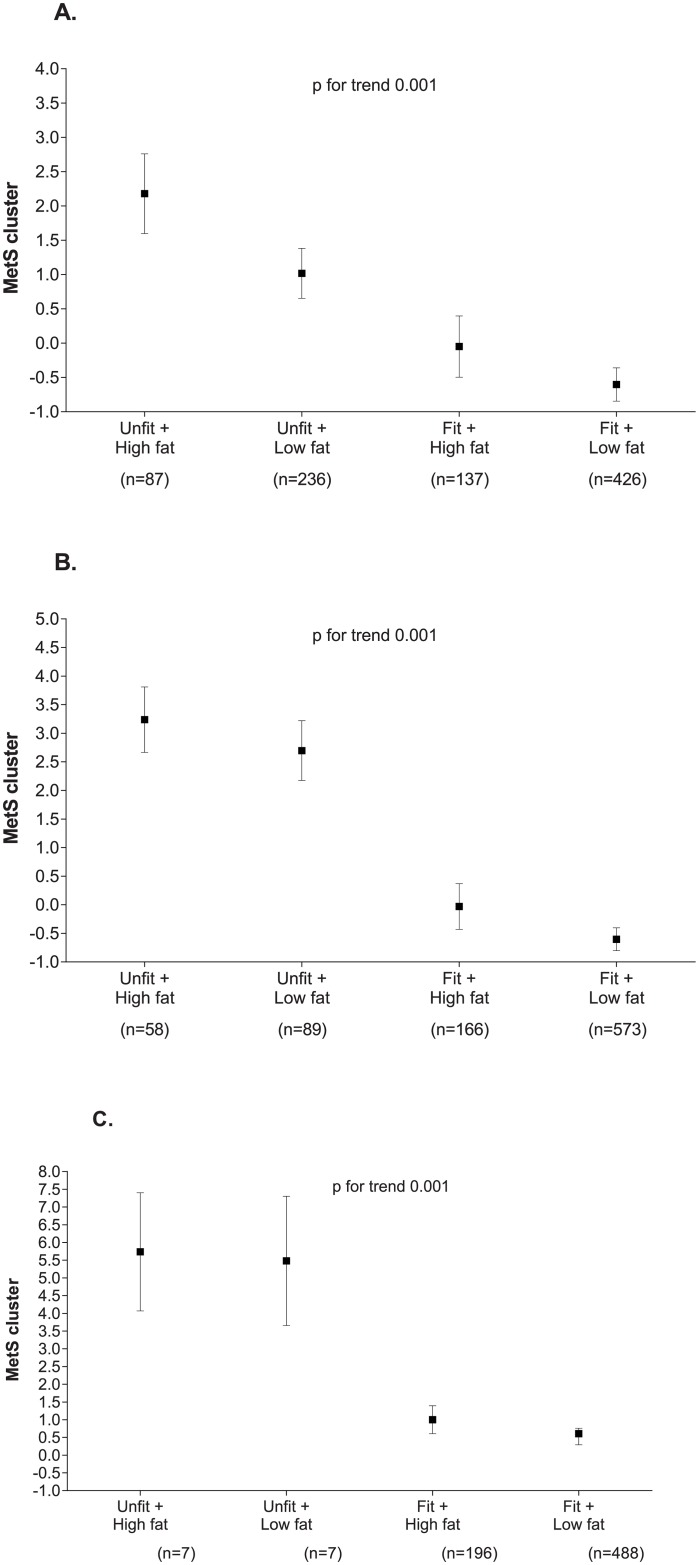
Combined effects of MF (unfit/fit) and fatness parameters on MetS cluster, adjusting for potential confounders. Estimated mean (dots) and 95% CIs (error bars) represent values after adjustment for age, sex, and lean mass (analysis of the covariance was used to test the group differences). A: FM, fat mass; B: BMI, body mass index; and C: VAT, visceral adipose tissue by bioelectrical impedance analysis.

The combined effect of MF and fatness parameters on the MetS cluster is presented in Fig 1. Overall, post-hoc analysis revealed that lower levels of the MetS cluster were associated with the healthiest profiles (fit + low fat) (p<0.001 in the four fatness parameters) compared with unfit and high fat counterparts.

### Mediation analysis

**Fig 2:**
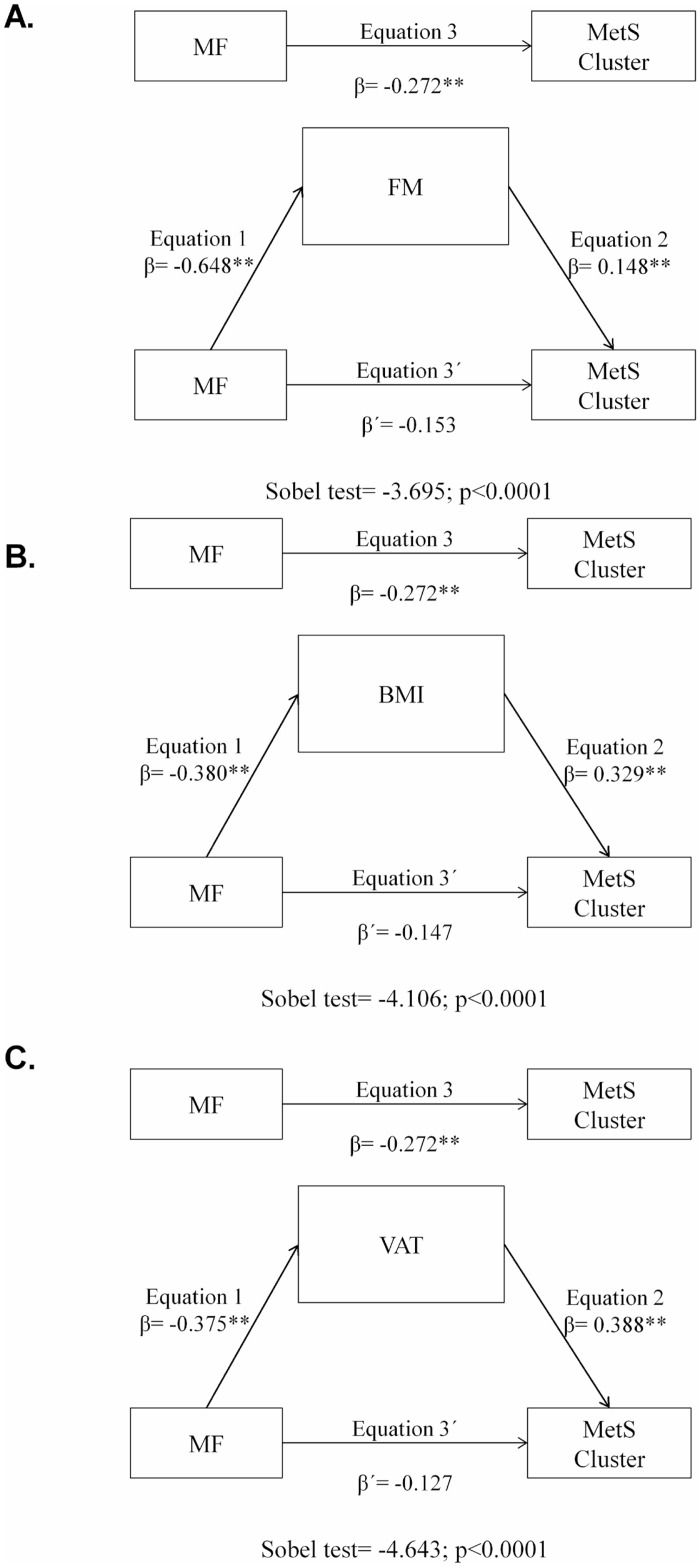
Fatness mediation models of the relationship between muscular fitness and MetS cluster, adjusting for potential confounders. A) FM, fat mass; B) BMI, body mass index; MF, muscular fitness; MetS cluster reflects a continuous score of the five MetS risk factors; and C) VAT, visceral fat, **p<0.001.

Overall, when we tested the mediating role of fatness parameters in the association between MF and MetS clustering, MF was negatively associated with fatness parameters (indirect). In the second equation, MF was negatively associated with MetS cluster (direct). Finally, in the third equation, when MF and fatness parameters were simultaneously included in the model, fatness was positively associated with MetS cluster (p < 0.001) (indirect), and although MF remained negatively associated with MetS cluster, these associations did not remain statistically significance. These results suggest that the effect of MF on MetS cluster was mediated by fatness parameters. Using the Sobel test for mediation adjusted by age, gender, lean mass, alcohol and tobacco intake, and geographical area, we found that 31% (z = -3.695; p <0.0001), 30% (z = -4.106; p <0.0001), 32% (z = -5.516; p <0.0001), and 31% (z = -4.643; p <0.0001) of the total effect of MF on MetS cluster was mediated by FM, BMI, and VAT, respectively (Fig 2), thus representing a partial mediation.

## Discussion

We have demonstrated that adults with good MF and lower fat profiles have significantly lower levels of MetS clustering compared to unfit adults with higher fat profiles. Furthermore, our results suggest a partial mediation of fatness parameters in the association of MF with MetS cluster, independent of gender and other confounders. These findings support those of another recent study which demonstrated that adjustment for waist circumference (from childhood) reduced the longitudinal effect for adult MetS by 17%–60% [29].

Both low levels of MF and increased adiposity have increasingly been recognized as important risk factors associated with MetS in adults [5, 12]. Regarding MF, skeletal muscle is the primary tissue for glucose and TG metabolism [30], and at the cellular level a limited number and size of myocytes have adverse metabolic consequences in terms of reduced glucose uptake and hyperglycaemia. This is partly due to the fact that transporter protein GLUT-4 expression at the plasma membrane is related to fibre volume in human skeletal muscle fibres [31]. Poor MF has been proposed to directly affect mortality through its association with increased disability and cardiometabolic disease. It has been hypothesized that one possible mechanism by which healthy MF exerts favorable health effects may be its capacity to reduce chronic low-grade inflammation. For example, Stenholm et al. [32] and Jurca et al. [33] provide strong evidence supporting the reduction of MF in the presence of catabolic biomarkers (C-reactive protein, Interleukin 6, Interleukin 1 receptor antagonist, Tumor Necrosis Factor-α), which increase oxidative stress, reduce muscle mass and cause losses of strength in middle-aged and elderly individuals. Another mechanism through which poor MF may influence health, for example, could be through modulation of increases in the enzyme lipoprotein lipase, which favors increased accumulation of TG and decreased HDL-c. In addition, low levels of MF increase insulin resistance and decrease glucose metabolism, and several studies have attempted to establish a relationship between MF, adiponectin levels (or the expression of adiponectin receptors), and reduce in insulin function.

There is significant interest in the independent and combined effects of physical fitness and fatness on health-related outcomes [34], but few studies have analyzed the combined effect on MetS. Overall, our study suggests that low fat and fit students had the best metabolic profiles compared to unfit and high fat counterparts; clearly highlighting that this combination poses a substantial health hazard. These results confirm those from other studies in university students [35, 36] and youth [37, 38]. A cross-sectional study by Kim et al. [36] examined 185 Korean university students and found that obese (expressed as BMI) and unfit (expressed as push-up, sit-up, and 1 mile run tests) subjects had higher risk of the MetS. Another study in 564 college students reported that being either overweight or less physically fit predisposed students to greater metabolic stress [35]. In adolescent populations, both studies [37, 38] showed that subjects who were in a high-fat/high-fit group showed significantly lower CVD risk factors than high-fat/low-fit group. These results suggest that improvement of physical fitness and reduction of fatness are both important factors for the prevention of MetS. Similar to the Sacheck et al. study [35], our results show that high levels of MF in the higher fat group attenuate MetS cluster, therefore, being physically fit can confer an added benefit to maintaining a healthy body composition. These results have been corroborated in overweight and Type 2 diabetes adults, reporting that when both fitness (expressed as cardiorespiratory fitness) and fatness (expressed as BMI) were examined together in the same model, fitness had a stronger association than fatness with several CVD factors such as glycated haemoglobin, ankle/brachial index, and Framingham risk score [39].

Our research has tested the “fat-but-fit” hypothesis (i.e., that it is fitness alone that confers health, irrespective of fatness) in young adults [40], and the results suggest that the influence of MF on MetS cluster was at least partially mediated by fatness, regardless of fatness parameters used. Although evidence has shown that young, fit adults with excess weight have lower metabolic risk than their unfit peers with excess weight [36], our mediation analysis does not fully support or completely refute the fat-but-fit hypothesis. In this sense, a study by Wijndaele et al. [41] found an independent association for MF with several individual metabolic syndrome risk factors in women, and most of these associations were only partially attenuate by central and general adiposity indicators (WC and BMI, respectively). Another cross-sectional study in 220 Japanese adults, aged 20–69 years old, demonstrated that MF was inversely associated with plasma glucose levels and clustered metabolic risk factors, independent of abdominal adiposity in women, but not in men [13]. Díez-Fernández et al. [42] suggested that, among school children, BMI mediates the association between MF and cardiometabolic risk, supporting our results from collegiate students. Even a randomized controlled trial [43] showed that although exercise improved fitness, the reductions in total and abdominal fatness and increase in lean body mass were more strongly associated with favorable changes in risk factors for cardiovascular disease in older adults. Therefore, the results of this study provide further evidence that fatness parameters are intermediate variables that need to be considered when analyzing the relationship between MF and cardiometabolic risk.

### Limitations

The primary limitation on our study was the cross-sectional design, which prevented us from making causal inferences. Thus, we are unable to deduce whether low MF (with or without excess adiposity) leads to higher risk of cardiometabolic abnormalities, or conversely, whether poor cardiometabolic profiles lead to declines in MF (i.e., reverse causation). Future longitudinal studies are needed to better understand how declines in muscle strength contribute to unhealthy metabolic profiles in young adults. Further, although fatness parameters were found to partially mediate the relationship between MF and MetS clustering, the determination coefficient of the regression models suggest that other competing factors such as physical activity and diet could also influence this relationship. Third, the use of field-tests such as standing broad jump and hand-grip strength for determination of MF are not ideal, but are valid and the most practical for a large study such as this. Fourth, fatness categories were based on international cut-offs (i.e., cut-off to body fatness unhealthy and cut-off to VAT unhealthy). Future research is needed to better describe the age- and sex-specific trajectories of strength as a predictor of comorbidities across the lifespan and, perhaps just as importantly, to apply robust analyses that can compartmentalize risk into hierarchical categories [44]. Finally, the thresholds for MetS are open to discussion and the values may vary based on these.

## Conclusions

The findings of this study indicate that efforts to improve MF in young adults may decrease MetS risk partially through an indirect effect on improvements to adiposity levels. As expected, college students with combined high fatness and low fitness had the poorest metabolic profiles. Thus, this study provides support for the importance of considering both fitness and fatness in contribution to MetS risk, and health care professionals should encourage physical activity participation to improve MF during this critical transition period between adolescence and young adulthood.
